# Trajectories of microsecond molecular dynamics simulations of nucleosomes and nucleosome core particles

**DOI:** 10.1016/j.dib.2016.04.073

**Published:** 2016-05-06

**Authors:** Alexey K. Shaytan, Grigoriy A. Armeev, Alexander Goncearenco, Victor B. Zhurkin, David Landsman, Anna R. Panchenko

**Affiliations:** a National Center for Biotechnology Information, NLM, NIH, Bethesda, MD 20894, United States; b Biology Department, Lomonosov Moscow State University, Moscow 119991, Russia; c Laboratory of Cell Biology, National Cancer Institute, NIH, Bethesda, MD 20892, United States

**Keywords:** Molecular dynamics, Nucleosome, Linker DNA, Histone, Histone tails

## Abstract

We present here raw trajectories of molecular dynamics simulations for nucleosome with linker DNA strands as well as minimalistic nucleosome core particle model. The simulations were done in explicit solvent using CHARMM36 force field. We used this data in the research article Shaytan et al., 2016 [1]. The trajectory files are supplemented by TCL scripts providing advanced visualization capabilities.

**Specifications Table**

**Table:** 

Subject area	*Biology*
More specific subject area	*Chromatin biology*
Type of data	*DCD files, PDB files, PSF files, TCL files*
How data was acquired	*Molecular dynamics simulations*
Data format	*raw*
Experimental factors	*Starting structure generated from PDB ID 1KX5*
Experimental features	*Explicit solvent, CHARMM36 force field*
Data source location	*–*
Data accessibility	via National Center for Biotechnology Information FTP server at ftp://ftp.ncbi.nih.gov/pub/panch/Nucleosome/MD

**Value of the data**

• Provides an atomic level data about nucleosome dynamics at microsecond timescale.
• Allows understanding the DNA dynamics in nucleosomes and nucleosome core particles.
• Shows interaction patterns of flexible histone tails with the DNA molecule.
• Can serve as a benchmark for further molecular dynamics (MD) simulations of nucleosomes.

## 1 Data

This data article presents microsecond molecular dynamics simulation trajectories which were analyzed in detail in Ref. [1]. Table 1 lists the simulated system names, short description and corresponding subfolder names under which the data was archived at the following URL ftp://ftp.ncbi.nih.gov/pub/panch/Nucleosome/MD. The trajectory files are presented in DCD file format, a corresponding topology PSF file and PDB file with the coordinates of the starting nucleosome structure are provided, as well as a TCL script to visualize the trajectory in VMD. Table 2 lists files with the short descriptions. Water molecules and ions are not included in the trajectory files. Each trajectory file contains 10,000 frames spanning one microsecond simulation time. Trajectories can be conveniently viewed in VMD by using the following command: vmd –e view.tcl.

## 2 Experimental design, materials and methods

### 2.1 Initial model construction

As a starting point we used high resolution (1.9 Å) X-ray crystal structure of nucleosome core particle formed by recombinant variants of *X. laevis* canonical core histones and modified human α-satellite DNA (PDB ID 1kx5 [2]). To create a full nucleosome (FN) model with the linker DNA segments, a straight 20 bp long B-DNA duplex (AGTC)_5_ was constructed using the NAB software [3]. The linker DNA sequence is balanced in the number of flexible and rigid base pair steps [4]. It was attached to the core DNA at both ends of NCP. One of the H3 histone tails was slightly rotated to avoid steric clashes with the linker DNA (ψ angle of Lys36 was set to −35°). The minimalistic model of NCP (NCPm) was obtained from the same crystal structure by clipping histone tails at the sites specified in Figure 2 of Ref. [1] by triangles. All models were explicitly solvated in a rectangular box with a minimum distance between the solute and the box boundaries of 20 Å. Sodium ions were added to the system for neutralization, and then additional sodium and chloride ions were added at a concentration of 150 mM with respect to the volume of water. Crystallographic water molecules were retained in the system, while all crystallographic ions were removed. Protonation states of amino acids were assigned based on their solution pK values at neutral pH, histidine residues were considered neutral and protonated on ε-nitrogens.

### 2.2 Simulation protocols

The CHARMM36 force field was used for DNA and protein [5], [6], TIP3P parameters for water molecules and adjusted ion parameters from Luo and Roux [7]. See detailed discussion of force field parameters in Ref. [1].

The simulation systems were prepared with the VMD program [8] and MD simulations were performed with the NAMD 2.9 package [9]. Langevin dynamics with 2 fs integration step, damping parameter of 0.5 ps^−1^ and *T*=310 K were used as means to perform constant temperature simulations. Pressure coupling was implemented via Langevin piston method and set to 1 atm. Simulations were performed with the rigid covalent bonds and Van der Waals interactions were gradually switched off over the distance between 10 and 12 Å. Electrostatic calculations employed PME method with grid spacing of 1 Å, cubic interpolation, 12 Å real space cutoff and direct space tolerance of 10^−6^. Periodic boundary conditions were used. To remove the nucleosome diffusion, slight constraints of 0.003 kcal/mol/A^2^ were applied to C-α atoms of H3 histone folds (residue numbers 64-78, 86-114, 121-131). To avoid base pair fraying at DNA ends in NCPm simulation a restricting artificial wall potential was used to keep the distance between the centers of mass of bases in the terminal base pairs within 120% of the initial.

All systems were initially subjected to energy minimization and initial equilibration via the following protocol: (i) 1000 steps of energy minimization with all protein molecules, DNA and crystallographic waters fixed, (ii) 10,000 minimization steps without atom fixation, (iii) four rounds of 200 ps simulations with elastic constraints on C-alpha atoms of protein and N1, N9 atoms of DNA bases which are gradually relaxed as follows: 300->150->30->0.9 kcal/mol/A^2^.

The production simulations were then performed up to the simulation time of 1 µs. The trajectory frames were saved every 100 ps. We run simulations in parallel on high performance computer clusters/supercomputers using effective parallelization available in NAMD. The simulation speed varied depending on the simulated system, number of CPUs and machine architecture. As a reference, the FN model system was simulated in parallel on 384 CPU cores for 120 days progressing at a pace of ~8 ns/day.

## Figures and Tables

**Table 1:** Simulated systems and sub-folder names.

Simulated system code (see Ref. [1] for details)	System description	Sub-folder name
FN	Full nucleosome together with linker DNA and full-length histone tails at physiological ionic strength	FN_model
NCPm	Minimalistic NCP model: no linker DNA, truncated histone tails	NCPm_model

**Table 2:** Data files provided for every simulation.

File name	Description
only_nucl_init.pdb	Initial conformation of nucleosome before equilibration and simulations
only_nucl_init.psf	psf topology file
md.dcd	1 μs MD trajectory saved every 100 ps
view.tcl	TCL script for VMD that can be used to view the trajectory
