# Supplementary material for: Trajectories of microsecond molecular dynamics simulations of nucleosomes and nucleosome core particles
Source: Data Brief. 2016 May 6;7:1678–81. doi: 10.1016/j.dib.2016.04.073 (PMC4872717; doi:10.1016/j.dib.2016.04.073)
Supplement: Supplementary file 1 — Supplementary material [file mmc1.docx]

Conflict of interest:none
